# The Importance of Lake Sediments as a Pathway for Microcystin Dynamics in Shallow Eutrophic Lakes

**DOI:** 10.3390/toxins7030900

**Published:** 2015-03-18

**Authors:** Haihong Song, Liah X. Coggins, Elke S. Reichwaldt, Anas Ghadouani

**Affiliations:** Aquatic Ecology and Ecosystem Studies, School of Civil, Environmental and Mining Engineering, the University of Western Australia, 35 Stirling Highway, M015, Crawley, WA 6009, Australia; E-Mails: haihong.song@research.uwa.edu.au (H.S.); liah.coggins@uwa.edu.au (L.X.C.); elke.reichwaldt@uwa.edu.au (E.S.R.)

**Keywords:** cyanobacterial bloom, microcystins, cyanotoxins, cyanobacterial biomass, sediments, spatial variability, temporal variability

## Abstract

Microcystins are toxins produced by cyanobacteria. They occur in aquatic systems across the world and their occurrence is expected to increase in frequency and magnitude. As microcystins are hazardous to humans and animals, it is essential to understand their fate in aquatic systems in order to control health risks. While the occurrence of microcystins in sediments has been widely reported, the factors influencing their occurrence, variability, and spatial distribution are not yet well understood. Especially in shallow lakes, which often develop large cyanobacterial blooms, the spatial variability of toxins in the sediments is a complex interplay between the spatial distribution of toxin producing cyanobacteria, local biological, physical and chemical processes, and the re-distribution of toxins in sediments through wind mixing. In this study, microcystin occurrence in lake sediment, and their relationship with biological and physicochemical variables were investigated in a shallow, eutrophic lake over five months. We found no significant difference in cyanobacterial biomass, temperature, pH, and salinity between the surface water and the water directly overlying the sediment (hereafter ‘overlying water’), indicating that the water column was well mixed. Microcystins were detected in all sediment samples, with concentrations ranging from 0.06 to 0.78 µg equivalent microcystin-LR/g sediments (dry mass). Microcystin concentration and cyanobacterial biomass in the sediment was different between sites in three out of five months, indicating that the spatial distribution was a complex interaction between local and mixing processes. A combination of total microcystins in the water, depth integrated cyanobacterial biomass in the water, cyanobacterial biomass in the sediment, and pH explained only 21.1% of the spatial variability of microcystins in the sediments. A more in-depth analysis that included variables representative of processes on smaller vertical or local scales, such as cyanobacterial biomass in the different layers and the two fractions of microcystins, increased the explained variability to 51.7%. This highlights that even in a well-mixed lake, local processes are important drivers of toxin variability. The present study emphasises the role of the interaction between water and sediments in the distribution of microcystins in aquatic systems as an important pathway which deserves further consideration.

## 1. Introduction

Microcystins are produced by certain species of cyanobacteria and are hazardous to humans and animals. Considered to be the most toxic group of cyanobacterial toxins, microcystins are inhibitors of specific protein phosphatases and can cause skin irritations, allergic reactions and gastroenteritis [1]. Humans are primarily exposed to microcystins via drinking water consumption and accidental ingestion of recreational water [2,3]. Recreational exposure by skin contact or inhalation to microcystins is now a recognised cause of a wide range of acute illnesses that can be life-threatening [4]. While microcystins have been reported across the world [5], the frequency of their occurrence in aquatic systems is expected to rise as a result of climate change [6,7,8,9,10]. To control the associated health risks, it is therefore essential to understand the fate of microcystins in aquatic systems.

**Figure 1:**
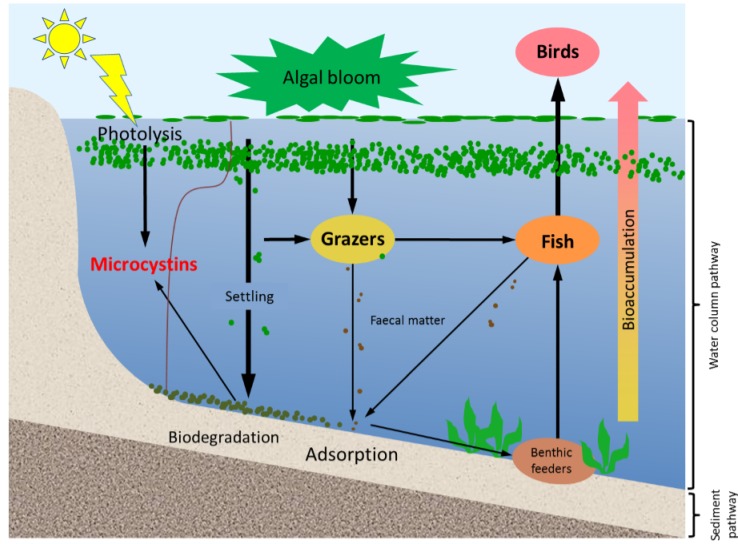
A schematic diagram showing the various pathways of microcystin dynamics during and following a bloom event. The sediment compartment plays a key role in release, adsorption and degradation of microcystins. Please note that stratification does not occur in all systems; especially shallow systems often have mixed water columns.

In aquatic systems, microcystins can be present not only in the water but also in sediments. They have been reported in a variety of sediments, with and without the occurrence of benthic cyanobacteria [11,12,13]. Microcystins in lake sediments have their origin in at least three main processes (Figure 1); including the lysis of cyanobacterial cells in sediments, transfer from the dissolved form to sediments by adsorption, and grazing by aquatic animals followed by the sinking of microcystin-containing faecal pellets [14]. Their occurrence in lake sediments is a potential hazard to aquatic animals and benthic organisms, for example, by altering the organisms’ metabolism [15,16]. In addition, the release of toxins that are loosely bound to the sediment might re-dissolve into the water column long after a bloom, and in absence of any visual indication of the presence of cyanobacterial blooms [17].

Studies on the distribution of total microcystins in sediments of aquatic systems are limited, likely due to technical difficulties in extracting total toxins from sediments [11]. An earlier study indicates that sediments contain two microcystin fractions: a loosely adsorbed and therefore “readily extractable” fraction, and a bound fraction [17]. The MMPB (2-methyl-3-methoxy-4-phenylbutyric acid) method is considered to be an effective analytical procedure for the quantification of total microcystins (free and bound) in lake sediments. However, this method requires a very low reaction temperature of −78 °C [11], which might be a limiting factor. Conversely, the loosely adsorbed microcystin fraction, which is possibly the more hazardous fraction for organisms due to its potential to easily re-dissolve back into the water column, is easier to extract, and some studies have focused on this fraction [14,18,19]. With conventional solvents, the extraction efficiency of loosely adsorbed microcystins strongly depends on the solvent type, the sediment characteristics, and the chemical structure of microcystins [12].

Microcystin concentrations in sediments have been shown to be highly variable on a temporal and spatial scale. High microcystin concentrations have been found in sediments during spring, followed by a decrease in the summer, when cyanobacterial biomass in the water was at a maximum [19]. Moreover, Chen *et al.* [14] found that the highest microcystin concentrations in sediments occurred during summer, when cyanobacterial biomass and microcystin production were at their maximum. Microcystins have been reported at different depths of sediments [11,20], with concentrations decreasing with increased depth of sediment in Lake Taihu, China [14].

Many factors can contribute to the variability of microcystins in lake sediments. Ihle *et al.* [19] reported that microcystin concentrations in the sediments of a shallow lake correlated to *Microcystis* biomass in the sediments. Similarly, microcystin concentrations in the sediments of the Nile River and irrigation canal sediments correlated to the total count of cyanobacteria, particularly *Microcystis* spp. and intracellular microcystins in the water [18]. The concentration of microcystins in sediments can also be influenced by the sedimentation of suspended particles with absorbed microcystins [21] and the adsorption of dissolved microcystins from the water [11,12,14,18,22,23]. Furthermore, organic matter content and particle size fraction (sand, silt and clay) [14,16,22,24], as well as the physicochemical parameters of lake water, such as temperature, salinity, and pH [24,25,26,27], can influence the sediment’s capacity to adsorb and degrade microcystins in aquatic systems. In shallow systems, wind-induced mixing of the water column and the associated redistribution of toxin-containing sediment potentially plays an important role in explaining the spatial distribution of toxins in the sediment.

Shallow lakes have a complex interplay between being stratified during times of high solar irradiation without wind and being completely mixed during periods of wind. Our study lake, Lake Yangebup, which is representative of a shallow water body, is a typical example of such a system with stratification only occurring during periods of wind speed <6 m/s [28]. In such well mixed water bodies, the horizontal distribution of allochthonous contaminants in sediments can be expected to differ very little, due to the highly dynamic nature of the sediment, which is being resuspended and redeposited during mixing events [28,29]. With autochthonous substances, such as cyanotoxins, the spatial distribution of toxins in the sediment depends on the location where it is produced and on the redistribution of sediments. Thus, in the absence of wind mixing, we could expect higher concentrations of toxins in the sediment at locations that have cyanobacterial blooms (Figure 1), while these horizontal differences should be reduced during wind-induced mixing events. The spatial distribution of toxins in the sediments of shallow lakes should therefore be the result of a complex interaction of biological and physicochemical processes, including wind-driven mixing.

To date, systematic studies on the relationship between the variability of microcystins in lake sediments and the environmental factors are lacking. While a number of studies have looked at the temporal variability of microcystins in sediments, the spatial variability of microcystins within aquatic systems, especially in shallow systems has not been investigated. This study aims to identify the spatial and temporal variability of microcystins in the upper layer of sediments in a shallow, eutrophic lake, and to determine the biological and physical parameters contributing to the variability. We hypothesised that the variability of microcystin concentration in lake sediments can be predicted by a combination of biological and physical parameters such as cyanobacterial biomass in the sediments, cyanobacterial biomass and microcystins in the water, and physicochemical parameters of lake water and the sediment characteristics.

## 2. Results

### 2.1. Temporal and Spatial Variability of Microcystin Concentration in Sediments

**Figure 2:**
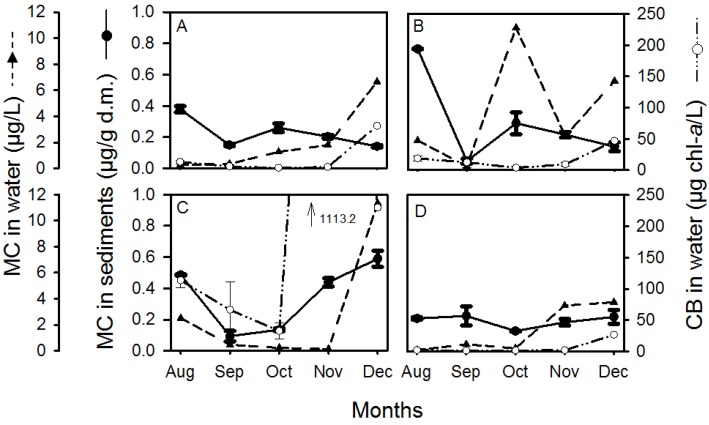
Microcystin concentration (MC) in sediments (●), total microcystin concentration in water (▲) and cyanobacterial biomass (CB) (○) in water as a function of time at sites 1–4 (**A**–**D**). CB in water is the calculated mean between CB in the surface water and the water directly overlying the sediment (overlying). Error bars represent one standard error (with *n* = 3 for MC concentration in sediments, and *n* = 2 for CB biomass in water). In November (panel C), cyanobacterial biomass was off the scale at 1113.2 µg chl-*a*/L, which is indicated by an arrow.

**Figure 3:**
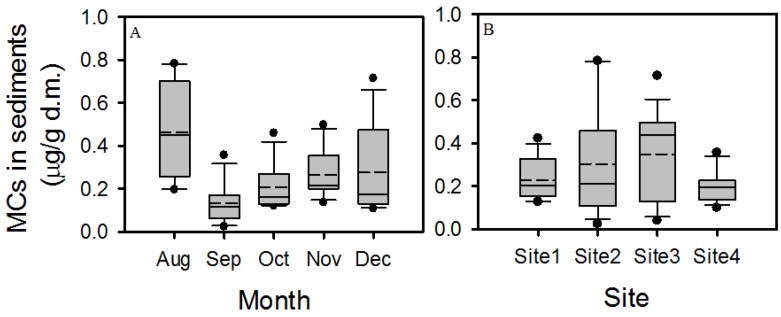
Spatial (**A**) and temporal variability (**B**) of microcystin concentrations in lake sediments. Boxes represent the 25th and 75th percentiles; solid lines within the boxes mark the median, dashed lines indicate the mean. Whiskers represent the largest and smallest observed values excluding the outliers. Filled circles represent outliers (>1.5 box lengths) (*n* = 15).

Microcystins were detected in all sediment samples, with their concentration ranging from 0.06 (S2; September) to 0.78 µg/g dry mass (d.m.) (S2; August) (Figure 2). The average microcystin concentration in the sediments decreased from August to September and increased from September to December (Figure 3A). The lowest average concentration was observed in September (0.13 µg/g d.m.) and the maximum average concentration was observed in August (0.46 µg/g d.m.).

The temporal variability of microcystin concentrations in sediments was different for each sampling site (Figure 2). There was a significant difference in microcystin concentration in the sediments between months at sites 1–3, but not at site 4 (Table 1) which had the smallest temporal variability of microcystins in sediments (Figure 2D). Furthermore, the temporal changes in microcystin concentrations in the sediment were similar at sites 1 and 2 (Figure 2), where they decreased from August to September, followed by an increase in October, and a decrease until December. In contrast, the microcystin concentration in the sediments at site 3 decreased from August to September but then increased until December, when the maximum concentration was observed (Figure 2).

**Table 1:** Statistical results for differences between sites (one-way ANOVA or Kruskal-Wallis ANOVA on ranks) and between months (repeated-measures ANOVA or Friedman repeated-measures ANOVA on ranks) of biological and physical parameters in the sediments and water. *** indicates *p* < 0.001; ** indicates *p* < 0.01; * indicates *p* < 0.05; d.f. = degrees of freedom; n.s. = not significant; d.m. = dry mass; chl-*a* = chlorophyll-*a*.

Biological and Physical Parameters	Differences between Months	Differences between Sites
Microcystins in sediments (µg/g d.m.)	F_(4,8)_ = 19.9 (Site 1) *** F_(4,8)_ = 36.2 (Site 2) *** F_(4,8)_ = 41.1 (Site 3) *** n.s. (Site 4)	H = 10.5, d.f. = 3 (August) * n.s. (September) n.s. (October) F_(3,8)_ = 21.5 (November) *** F_(3,8)_ = 21.4 (December) ***
Cyanobacterial biomass in sediments (µg chl-*a*/g d.m.)	F_(4,8)_ = 9.9 (Site 1) ** n.s. (Site 2) n.s. (Site 3) F_(4,8)_ = 3.9 (Site 4) *	H = 8.8, d.f. = 3 (August) * H = 9.5, d.f. = 3 (September) * n.s. (October) n.s. (November) H = 9.5, d.f. = 3 (December) *
Cyanobacterial biomass in surface water (µg chl-*a*/L)	χ^2^ = 12.2, d.f. = 4 *	H = 12.6, d.f. = 3 **
Cyanobacterial biomass in overlying water (µg chl-*a*/L)	χ^2^ = 13.6, d.f. = 4 **	n.s.
Cyanobacterial biomass-average (µg chl-*a*/L)	χ^2^ = 11.5, d.f. = 4 *	H = 11.3, d.f. = 3 *
Intracellular microcystin concentration in water (µg/L)	F_(4,12)_ = 10.9 ***	n.s.
Dissolved microcystin concentration in water (µg/L)	χ^2^ = 11.8, d.f. = 4 *	n.s.
Total microcystin concentration in water (µg/L)	F_(4,12)_ = 3.5 *	n.s.
Organic matter (%)	F_(4,12)_ = 31.7 ***	n.s.

The spatial variability of microcystin concentrations in sediments was different in each month. In August, microcystin concentration in sediments at site 4 was significantly lower than at site 2, while in November and December, microcystin concentration was significantly higher at site 3 than at any other site (Table 1; Figure 2). In September and October, no significant differences in microcystin concentration between sites were observed. The largest spatial variability of microcystins in sediments occurred in August, while the smallest occurred in September (Figure 3A). On average, the microcystin concentration in sediments was highest at site 3 and lowest at site 4 (Figure 3B).

### 2.2. Temporal and Spatial Variability of Biological and Physical Parameters

Cyanobacterial biomass in sediments varied significantly at sites 1 and 4 over time (Table 1), and was significantly different between sites in: (i) August, when biomass at site 3 was significantly higher than at site 4; (ii) September, when biomass at site 3 was significantly higher than at sites 1 and 4; and (iii) December, when biomass at site 1 was significantly higher than at site 4 (Table 1).

Cyanobacterial biomass in the water was not significantly different between the surface water layer and the water layer directly overlying the sediment (overlying water) (*t*-test; *t* = −1.23, d.f. = 44), indicating a mixed water column. Cyanobacterial biomass, averaged over the water column (surface + overlying samples), showed a similar temporal trend at all sites and was highest at site 3 (Figure 2), with an extremely high value of 1113.2 µg chl-*a*/L in November. There was a significant difference between sites in (i) cyanobacterial biomass in surface water (Table 1), with site 3 having a higher biomass than sites 1 and 4, and (ii) cyanobacterial biomass averaged over the water column (Table 1), with site 3 having a higher biomass than site 4. There was no difference between the sites for cyanobacterial biomass in the overlying water (Table 1). Cyanobacterial biomass in surface water, overlying water, and averaged over the water column was significantly different between months (Table 1). The average cyanobacterial biomass in the water column and the cyanobacterial biomass in the surface water were higher in December than October, while the cyanobacterial biomass in the overlying water was significantly higher in December than in September.

Total microcystin concentration (intracellular + dissolved) in surface water showed a similar trend at all sites with increasing concentrations towards December (Figure 2) and was significantly different between months (Table 1). Intracellular and dissolved concentrations in the water were both significantly different between months (Table 1), with December concentrations being different to all other months. There was no difference in intracellular, dissolved, or total microcystin concentration between sites (Table 1).

Organic matter content in sediment samples was different between months, with the percentage organic matter being significantly higher in December compared to all other months (Table 1). No significant difference in organic matter between sites was detected.

### 2.3. Correlation between Microcystins in the Sediments and Biological and Physical Parameters

Linear correlations (Pearson’s correlations) were calculated between microcystin concentration in sediments and the biological and chemical parameters studied (Table 2). Microcystin concentration in the sediments had weak but significant linear correlations with the concentration of intracellular microcystin in the water, total microcystin concentration in surface water, and cyanobacterial biomass in the water directly above the sediment (overlying).

**Table 2:** Pearson’s correlation values (R) between microcystin concentrations (MC) in the sediments (µg/g d.m.), biological and chemical parameters. CB_sedim_: cyanobacterial biomass in the sediments (µg chl-*a*/g d.m.); CB_overl_, CB_surf_, CB_average_: cyanobacterial biomass in the overlying, the surface water, or averaged over the two layers (µg chl-*a*/L); MC_intra_, MC_diss_, tMC_water_: intracellular, dissolved, and total microcystin concentration in the surface water (µg/L); OM: percentage (mass) of organic matter in the sediments; Sig. = *p*-value. All data were log transformed before analysis.

	CB_sedim_	CB_overl_	CB_surf_	CB_average_	MC_intra_	MC_diss_	tMC_water_	OM
**MC_sedim_**	−0.012	0.476	0.171	0.237	0.289	0.016	0.254	0.108
**Sig.**	0.929	0.000	0.193	0.068	0.025	0.906	0.050	0.418
***N***	60	60	60	60	60	60	60	58

### 2.4. Prediction of Microcystin Concentrations in Sediments from Biological and Physical Parameters

Assuming a well-mixed system, the concentration of microcystins in the sediments $\left(\text{c}\left(MC_{se\dim}\right)\right)$ could be best predicted by:

$\log c\left(MC_{sedim}\right)=\;5.784\;+\;0.229\log c\left(tMC_{water}\right)+\;0.230\log c\left(CB_{average}\right)-\;0.170\log c\left(CB_{sedim}\right)\;-\;7.28\log pH$, with c(*tMC_water_*) being the total microcystin concentration in the water, $\log c\left(CB_{average}\right)$ being the average cyanobacterial biomass in the water column, and $\text{c}\left(CB_{sedim}\right)$ being the cyanobacterial biomass in the sediment (*R^2^* = 0.211, *p* < 0.05, F_(4,55)_ = 3.68).

A second multiple linear regression analysis using explanatory variables expressing processes on smaller scales increased the *R^2^* to 0.517 (*p* < 0.05, F_(5,54)_ = 11.055):

$\log c\left(MC_{sedim}\right)=10.285\;+0.524\log c\left(\left(MC_{intra}\right)\;+1\right)+0.309\log c\left(CB_{overl}\right)-0.132\log c\left(CB_{sedim}\right)-11.402\log pH-0.655\log temperature$, with *c*(*MC_intra_)* being intracellular microcystins in the water, $\left(CB_{overl}\right)$ being cyanobacterial biomass in the overlying water, and $c\left(CB_{sedim}\right)$ being cyanobacterial biomass in the sediment.

## 3. Discussion

Microcystins were detected in all sediment samples in this study, including at site 4 where only a low level of cyanobacterial biomass occurred, indicating the wide presence of microcystins in the lake’s sediments. Similar to previous studies [30,31,32], we found that cyanobacterial blooms, dominated by toxic *Microcystis* spp., and microcystins in the water occurred most of the year in Lake Yangebup. Microcystins can therefore accumulate in the sediments as a result of its long-term exposure to cyanobacterial biomass at the bottom of the lake and microcystins in the water (Figure 1).

**Figure 4:**
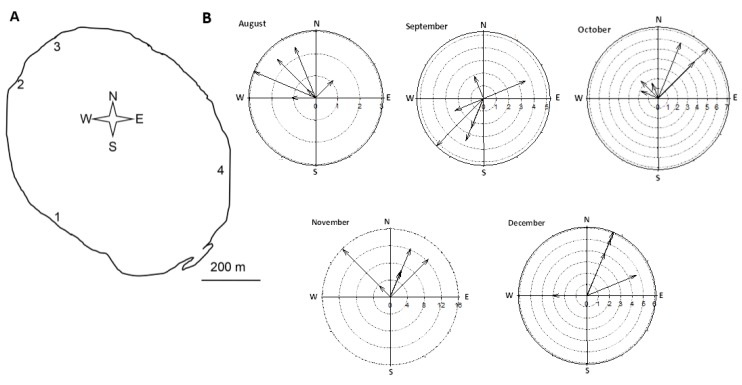
Study site and wind conditions. (**A**) Map of Lake Yangebup with sampling sites; (**B**) Wind speed and direction for the sampling days and the two antecedent days for each month. Wind measurements were taken at 9 am and 3 pm of each day, resulting in 6 measurements for each month. Please note different axes for wind speeds.

The occurrence of microcystins in lake sediments associated with cyanobacterial blooms has been reported in earlier studies [11,12,14,18,19]. Microcystin concentrations in the sediment depend on a number of factors, including toxin production within the cyanobacterial community, and the biomass of toxic cyanobacteria in the sediment and in the water column [18,19]. Once the toxins that are produced within the water column reach the sediment, physical, chemical, and biological factors that influence the sediment’s capacity to adsorb and degrade microcystins play an important role. The spatial variability of toxins in the sediment of a lake will therefore be the result of a complex interaction between the toxin producing processes in the water column, the physico-bio-chemical processes in the sediment, and a redistribution of toxin containing sediment. The latter is of extreme importance in shallow lakes, where wind is responsible for de-stratification of the water column and re-distribution of sediments [28,29]. An earlier study in Lake Yangebup showed that there is complete mixing of the water column at wind speeds >6 m/s with intermittent stratification at lower wind speeds, which can overturn within a few days [28]. Although wind speed was partially <6 m/s during our study (Figure 4B), these earlier results from Arnold and Oldham [28], in addition to the fact that we did not find a difference between the surface water layer and the water layer directly above the sediment (overlying) in cyanobacterial biomass, temperature, salinity and pH, indicates that Lake Yangebup is a typical representative of a shallow lake, that can be considered a mixed system due to wind effects and diurnal convectional cooling of the water.

Interestingly, microcystin concentration and cyanobacterial biomass in the sediment was different between sites in three out of five months. This indicates that site specific processes are important for these variables, and that a re-distribution of toxin and cell containing sediment by wind-mixing might not have been complete. At the same time, we did not find a difference in the sediment toxin concentration between sites in September, when site 3 had a significantly higher cyanobacterial biomass in the water than all other sites, and differences in the sediment toxin concentration in November also did not coincide with different cyanobacterial biomass in the water column. This shows that some level of mixing is important in explaining the distribution of microcystins in the sediment. We can therefore conclude that local and wind-mixing processes are both important factors affecting the concentration of microcystin in the sediment.

We could explain the variability of microcystin concentration in the sediments by a combination of environmental variables, namely total microcystin concentration in the water, cyanobacterial biomass in the water, cyanobacterial biomass in the sediment and pH. These factors have also been shown to be of importance in previous studies, where significant and close relationships between the concentration of microcystins in sediments and intracellular microcystins or cyanobacterial biomass in the water column and the sediment have been reported [18,19]. The microcystins produced in the cyanobacteria in the water column can reach the sediment via a number of pathways, including settlement of cyanobacterial cells with subsequent cell lysis, and the adsorption of dissolved toxins to the sediment (Figure 1). The percentage of explained variability could be increased significantly by forcing variables indicative of smaller scale processes, such as cyanobacterial biomass in the overlying water, cyanobacterial biomass in the surface water, and intracellular and dissolved microcystin concentrations into the model. This clearly highlights again that, although Lake Yangebup is a mixed system, local processes are important to explain the local microcystin concentration in the sediment. Furthermore, it emphasises the importance of the link between processes in the water column in explaining the microcystin concentration in sediments [14,21].

The multiple linear regression analysis shows that microcystin concentration in sediments decreased with increasing pH and temperature. This could be due to the fact that higher temperatures increased the sediments capability to biodegrade microcystins, aiding the removal of these toxins from aquatic systems [25,33], and due to higher temperatures leading to an increased metabolic activity of the microcystin-degrading bacteria. In addition, previous studies have also shown that lower pH increases the capacity of sediments to adsorb microcystins [24,26,27], by influencing the surface charge heterogeneity of microcystins and sediment [17].

The temporal variability of microcystins in the lake’s sediments was site specific, and the spatial variability in their concentration was different in each sampling month. Site 3 experienced a massive development of cyanobacterial biomass in November and December, and this possibly resulted in the increase of toxin concentration in the sediment at this site. On average, cyanobacterial biomass in the water was lowest at site 4 and highest at site 3. Wind is known to be a main driver of horizontal distribution of cyanobacterial biomass in lakes [34]. Wind speed and direction was variable in our study, however site 3 was the most downwind site in all months except September (Figure 4B). Therefore, it is likely that wind is the reason why we detected the highest biomass at this site.

The physical and chemical properties of sediments, for example, organic matter content and particle size fraction (sand, silt, and clay), will affect the sediment’s capacity to adsorb and degrade microcystins [14,16,22,23,24]. Mohamed *et al.* [18] observed that in the Nile River, the capacity of sediments to adsorb microcystins was significantly correlated to their clay and organic matter content. Wu *et al.* [27] found that the adsorption process for microcystins to sediments mainly depended on the sediment’s organic matter content. The significance of organic matter and clay in the binding and degradation of microcystins to soils and sediments has been reported in many other studies [24,35,36,37,38,39,40]. In our study, no clay was present in the sediments and no significant correlation between the concentration of microcystins and organic matter content in sediments was observed, probably due to only minor differences in the organic matter content of the samples.

## 4. Experimental Section

### 4.1. Study Site

This study was conducted at Lake Yangebup in Western Australia (32°6'40"S, 115°50'00"E). The field permit was provided by the City of Cockburn, Western Australia. The field study did not involve endangered or protected species. Lake Yangebup is a shallow, eutrophic, permanent lake with approximately 68.4 ha of open water and a mean water depth of 2.5 m [28]. Total phosphorus and total nitrogen in the water column of Lake Yangebup was previously reported to be 0.49–6.98 µM and 0.14–0.37 mM, respectively [32,41]. Toxic cyanobacterial blooms, dominated by *Microcystis* spp., frequently occur in this lake, and microcystins are present in the water for most of the year [30,31,32]. In a previous study in 2008–2009 [32,42], the range of concentration of microcystins in the water column was 1–80 µg equivalent microcystin-LR/L. Lake Yangebup is a groundwater through-flow lake that receives groundwater from its eastern side and discharges it towards the west. An earlier study, over 20 months using a weather station and a thermistor chain in the water column with sensors every 40 cm, showed that Lake Yangebup is mixed without stratification when wind speed is >6 m/s [28]. Sediment resuspension and redistribution during periods of wind-mixing has been hypothesised to be an important factor responsible for the horizontal distribution of sediments and contaminants in this and a neighbouring lake [28,29]. Lake Yangebup is surrounded by patches of fringing vegetation and an earlier investigation has reported the presence of a flocculate sediment layer, consisting mainly of dead and decaying organic matter covering a more consolidated sediment layer [28].

### 4.2. Sampling Design

**Figure 5:**
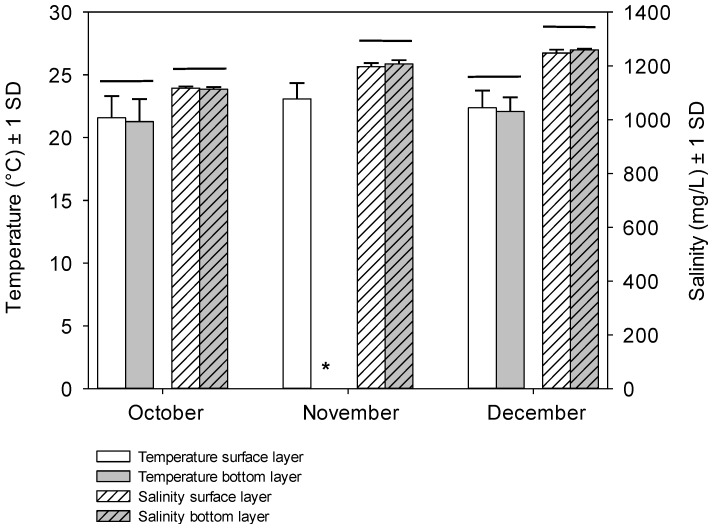
Mean (±1 SD) temperature (°C) and salinity (mg/L) in the surface and overlying water layers measured at the four sites in October to December in Lake Yangebup during this study. * = missing data; horizontal line indicates that no significant difference between data were detected (student *t*-test).

Samples were taken monthly between August and December 2010, from four shore sites of Lake Yangebup with coordinates for sites 1–4 being 32°07'21"S, E115°49'45"; 32°07'07"S, 115°49'31"E; 32°6'50"S, 115°49'43"E; 32°07'11"S, 115°50'07"E, respectively (Figure 4A). Sampling sites were chosen based on their accessibility and geographic locations with the aim to distribute our sampling sites around the whole lake. All samples were taken between 7:30 am and 1:30 pm from water with a depth of 0.6–0.7 m and were stored on ice, in the dark, for transport to the laboratory. Temperature, pH, salinity were taken *in situ* from 10 cm below the water surface (referred to as “surface water”) in all months, and additionally directly above the sediments (referred to as “overlying water”) in October to December with probes (TPS WP-81); the average values of these parameters are given in Table 3. Dissolved oxygen was only measured at a depth of 10 cm (TPS-DO2). At each site, measurements for both surface water and overlying water were taken above the points where sediment samples were collected. There was no significant difference in temperature and salinity between the surface and the overlying water layer during the sampling period (student *t*-test; Figure 5), indicating that the water column was well mixed. Dissolved and intracellular toxins in surface water samples and cyanobacterial biomass in surface water and overlying water samples were quantified in the laboratory. For each sampling site, three sediment samples (0–4 cm) were collected using a transparent, polycarbonate sediment corer with a stainless-steel cutter (50 mm in diameter). Sediments in each sample were thoroughly mixed for homogenous quantification of cyanobacterial biomass, microcystin concentrations, and physicochemical properties. Wind direction and speed data came from the Australian Bureau of Meteorology’s monitoring station at Jandakot Airport, which is 3 km from Lake Yangebup (Figure 4B).

**Table 3:** Physicochemical parameter means in each sampling month in Lake Yangebup. - indicates no measurement due to failure of the probe. DO = dissolved oxygen.

Month	Temperature (°C)	Salinity (mg/L)	DO (%)	DO (mg/L)	pH
August	16.4	946	44.9	4.9	8.4
September	18.9	947	54.9	5.0	8.6
October	21.4	1115	63.3	5.6	8.4
November	23.1	1202	-	-	8.6
December	22.2	1254	123.9	11.1	9.4

The mean daily air temperature, recorded at the nearest weather station to Lake Yangebup, taken over the 14 days prior to the sampling day, was used to represent surface water temperature in the data analysis. This was chosen instead of direct water temperature measurements, as water temperature strongly depends on the time of day sampling takes place. Earlier studies reported a good correlation between water temperature in shallow lakes and air temperature, and found that microcystin concentration in the water could be best predicted by mean daily air temperature of the preceding 14 days [43].

### 4.3. Sediment Analysis

The particle size of sediment samples was measured using a method adapted from Bowmanand Hutka [44]. The adaptations included heating the samples for a quicker digestion of the organic matter and removing the hydrogen peroxide by sequential dilution instead of evaporation. The particle size distribution analyser used was the Malvern Mastersizer 2000 with a Malvern Hydro 2000G automated wet dispersion accessory. Organic matter, measured by loss-on-ignition, was analysed according to Heiri *et al.* [45] but with samples dried in an oven at 110 °C overnight instead of freeze drying. The organic matter content of the sediment samples in Lake Yangebup ranged from 0.55% to 6.17%. With respect to particle size distribution, all sediment samples were sand, consisting of coarse and fine sand particles with a coarse sand content of 88% ± 10.12%. The particle size distribution for sediment samples at each sampling site is given in Table 4.

**Table 4:** Particle size fractions of sediment samples at each sampling site.

Sampling Sites	Clay % (<2 µm)	Silt % (2–20 µm)	Fine Sand % (20–200 µm)	Coarse Sand % (200–2000 µm)	Total Sand %
Site 1	0	0	13.12 ± 6.45	86.88 ± 6.45	100
Site 2	0	0	21.05 ± 2.71	78.95 ± 2.72	100
Site 3	0	0	10.66 ± 2.88	89.40 ± 2.88	100
Site 4	0	0	3.22 ± 0.87	96.78 ± 0.87	100

### 4.4. Analysis of Dissolved Microcystins in the Water

Surface water samples, 900–1000 mL for each water sample, were first filtered through pre-combusted GF/C filters (Whatman). Each filtrate was then applied to a solid phase extraction (SPE) cartridge (Oasis HLB 6 cc/500 mg, Waters, Rydalmere, NSW, Australia) with a flow rate <10 mL per minute for cleaning and concentration. The cartridge was then washed with 5 mL of 10% and 20% (*v/v*) methanol, in sequence, before blowing a constant air flow through the cartridge for drying. Microcystins were eluted from the cartridge with 5 mL of 100% methanol +0.1% trifluoroacetic acid (*v/v*). The elutes were dried with nitrogen gas at 45 °C before being re-dissolved in 1 mL of 30% acetonitrile (*v/v*) and analysed by high-performance liquid chromatography (HPLC) with a photodiode detector (1.2 nm resolution; Alliance 2695, Waters Corporation, Rydalmere, NSW, Australia) and an Atlantis^®^ T3 separation column (4.6 × 150mm i.d, 3 µm, 100 Å; Waters Corporation). The HPLC gradient was identical to Lawton *et al.* [46] but with a maximum of 100% acetonitrile and, therefore, a longer run time of 37 min. Column temperature was 37.5 ± 2.5 °C and the limit of detection was 1.12 ng. Our limit of quantification was 3× the limit of detection. Peaks that showed a typical microcystin absorption spectrum with a maximum at 238.8 nm were quantified by comparing the peak area with the area of a known standard (microcystin-LR; Sapphire, Sydney, Australia).

### 4.5. Analysis of Intracellular Microcystins in the Water

Water samples were filtered on pre-combusted and pre-weighed GF/C filters (Whatman). The filters were dried at 60 °C, for 24 h, before being re-weighed to quantify biomass and then stored at −21 °C until microcystin extraction. Before the extraction of microcystins, the dry filters were thawed and re-frozen three times. The extraction was achieved with 6 mL of 75% methanol (*v/v*) per filter. Filters were sonicated on ice for 25 min, followed by gentle shaking for another 25 min. The extracts were then centrifuged at 3273× *g* (Allegra X-12 Series; Beckman and Coulter Inc., Lane Cove, NSW, Australia) for 10 min at room temperature. Extracts were collected and filters were extracted two more times. After the three extractions, the supernatants were combined and diluted with Milli-Q water from 75% to 20% methanol (*v/v*) before they were applied to SPE cartridges (Oasis HLB 6 cc/500 mg, Waters, Australia). The subsequent analysis procedure was identical to that used for the dissolved microcystin analysis.

### 4.6. Analysis of Microcystins in the Sediments

The method used for quantification of microcystins in the sediments was adapted from Babica *et al.* [12]. In short, each freeze-dried sediment sample, 2.0–2.2 g (dry mass), was extracted twice with 20 mL of 5% acetic acid in methanol containing 0.2% *v/v* trifluoroacetic acid (TFA), using an ultrasonic bath for 30 min. After each extraction, the sample was centrifuged for 10 min at 3273× *g* (Allegra X-12, Beckman Coulter) and the supernatants from both extractions were combined, diluted with Milli-Q water from 95% to 20% methanol (*v/v*) and then applied to SPE cartridges (Oasis HLB 6 cc/500 mg, Waters, Australia). The subsequent analysis procedures, such as the cleaning and concentration of microcystins using SPE cartridges and the quantification of microcystins with HPLC, were identical to those used for the dissolved microcystin analysis. In a preliminary laboratory test, we determined the recovery rate for easily extractable microcystin-LR in sediment samples using this method was above 87% (data not shown).

Throughout this study, we refer to the total concentration of microcystin variants per sample as microcystin concentration. Total microcystin concentration (intracellular + dissolved) was expressed as micrograms (microcystin-LR mass equivalents) per litre water, intracellular microcystin concentration was expressed as micrograms (microcystin-LR mass equivalents) per gram cyanobacterial dry mass, and the total concentration of microcystin variants per sediment (easily extractable microcystins) was micrograms (microcystin-LR mass equivalents) per gram dry sediments.

### 4.7. Cyanobacterial Biomass in the Sediments and Water

The cyanobacterial biomass of each water sample was measured in the laboratory with a bench top version of the FluoroProbe (BBE Moldaenke, Schwentinental, Germany) as µg chl-*a*/L [47,48], for which chorophyll-*a* is a proxy for cyanobacterial biomass. The validation of the measurement of total chlorophyll-*a* with the Fluoroprobe against the values obtained from samples extracted according to standard methods [49] was described in Sinang *et al.* [32]. A strong linear correlation was found between the chlorophyll-*a* concentration measured with FluoroProbe and the chlorophyll-*a* concentration measured with the standard method (*R^2^* = 0.94, *N* = 32, *p* < 0.05). Sediment samples were washed with tap water until no cyanobacteria colonies in the sediments could be observed microscopically. The tap water was filtered through gauze (63 µm) to capture these colonies, which were then transferred with 30 mL of deionized water into a 100 mL glass bottle. This washing procedure was repeated twice and the cyanobacterial colonies were combined in a total of 100 mL deionized water. The concentration of cyanobacterial biomass in this bottle was quantified using the bench top version of the FluoroProbe as µg chl-*a*/L.

### 4.8. Statistical Analyses

Differences between sites were analysed using a one-way analysis of variance (ANOVA) when the data was normally distributed, and a Kruskal-Wallis ANOVA on ranks with a Bonferroni *t*-test when the data failed the normality test. Differences between months were analysed with a repeated-measures ANOVA with a Bonferroni *t*-test when the data was normally distributed, and a Friedman repeated-measures ANOVA on ranks with a Bonferroni *t*-test when the data failed the normality test. Pearson’s correlations were calculated to identify the correlation between the concentration of microcystins in lake sediments and biological and physical parameters.

Two multiple linear regression analysis (backward) were carried out on the log transformed data to identify models that best explained the variability in the concentration of microcystins in the sediments. The first analysis included the following explanatory variables, which are representative for systems that are vertically well-mixed: cyanobacterial biomass in the sediment (c(CB_sedim_); µg chl-a/g d.m.); total microcystin concentration in the water (c(tMC_water_)); µg/L); average cyanobacterial biomass in water (c(CB_average_); µg/L); and pH and temperature. The second analysis included explanatory variables, which present processes on a smaller local scales: cyanobacterial biomass in the sediment (c(CB_sedim_); µg chl-a/g d.m.); intracellular microcystin concentration in the water (c(MC_intra_)); µg/L); dissolved microcystin concentration in the water (c(MC_diss_)); µg/L); cyanobacterial biomass in overlying (c(CB_overl_); µg/L) and surface water (c(CB_surf_); µg/L); and pH and temperature. We used 0.05 as the probability of F-to-remove. Prior to the statistical analyses, the linearity of the microcystin concentration in sediments and the biological and physical parameters hypothesised to control microcystin variability were tested using scatterplots. Both dependent and independent variables were log-transformed and checked for normality using a Kologorov-Smirnov test. Statistical analyses were conducted in SigmaPlot 12.0 (Systat Software Int., San Jose, CA, USA) and IBM SPSS 21.0 (SPSS Inc., Chicago, IL, USA). Significance levels were set as *p* < 0.05 unless stated otherwise.

## 5. Conclusions

For the management of bloom-prone water bodies, it is important to holistically understand the fate of microcystins in the aquatic systems. Current studies and policies focusing on microcystins in aquatic systems almost exclusively concentrate on the water column, while sediments are mostly neglected. However, there is strong evidence from this and earlier studies that microcystins are widely present in sediments. In our study, the spatial variability of microcystins concentrations in sediments was different in each sampling month and their temporal variability was site specific. This highlights the fact that both local processes and lake-wide mixing contribute to the spatial distribution of microcystins in the sediment. It is interesting to note that, although we used a fairly small dataset, we could statistically identify drivers of the microcystin concentration in the sediment. We found that a significant part of the variability of the microcystin concentration in the sediments could be explained by a combination of variables in the water column, such as total microcystins in the water, cyanobacterial biomass in water, pH, and temperature. This illustrates the significance of the interaction between water and sediments in the distribution of microcystins in the sediment. In conclusion, our study highlights that cyanobacterial toxins in the sediment could pose a potential ecological hazard for the system, and that water management authorities should include sediments when assessing the potential health risks from microcystins in aquatic systems. This is especially important for shallow lakes that have an intimate link between the sediment and the water column. Further studies are required to study the role of local versus mixing processes for the microcystin distribution within sediments. In addition, long-term studies are essential to identify any seasonal patterns of sediment as a pathway for cyanotoxin dynamics.

## References

[1] Žegura B., Štraser A., Filipič M. (2011). Genotoxicity and potential carcinogenicity of cyanobacterial toxins—A review. Mutat. Res..

[2] Chorus I., Falconer I.R., Salas H.J., Bartram J. (2000). Health risks caused by freshwater cyanobacteria in recreational waters. J. Toxicol. Env. Health B.

[3] Funari E., Testai E. (2008). Human health risk assessment related to cyanotoxins exposure. Crit. Rev. Toxicol..

[4] De la Cruz A.A., Antoniou M.G., Hiskia A., Pelaez M., Song W.H., O’Shea K.E., He X.X., Dionysiou D.D. (2011). Can we effectively degrade microcystins?-Implications on human health. Anti Cancer Agent Med. Chem..

[5] Zurawell R.W., Huirong C., Burke J.M., Prepas E.E. (2005). Hepatotoxic cyanobacteria: A review of the biological importance of microcystins in freshwater environments. J. Toxicol. Environ. Health B.

[6] Moore S., Trainer V., Mantua N., Parker M., Laws E., Backer L., Fleming L. (2008). Impacts of climate variability and future climate change on harmful algal blooms and human health. Environ. Health Glob..

[7] Paerl H.W., Huisman J. (2009). Climate change: A catalyst for global expansion of harmful cyanobacterial blooms. Environ. Microbiol. Rep..

[8] El-Shehawy R., Gorokhova E., Piñas F.F., del Campo F.F. (2012). Global warming and hepatotoxin production by cyanobacteria: What can we learn from experiments?. Water Res..

[9] Reichwaldt E.S., Ghadouani A. (2012). Effects of rainfall patterns on toxic cyanobacterial blooms in a changing climate: Between simplistic scenarios and complex dynamics. Water Res..

[10] Paerl H.W., Paul V.J. (2012). Climate change: Links to global expansion of harmful cyanobacteria. Water Res..

[11] Tsuji K., Masui H., Uemura H., Mori Y., Harada K. (2001). Analysis of microcystins in sediments using MMPB method. Toxicon.

[12] Babica P., Kohoutek J., Blaha L., Adamovsky O., Marsalek B. (2006). Evaluation of extraction approaches linked to ELISA and HPLC for analyses of microcystin-LR, -RR and -YR in freshwater sediments with different organic material contents. Anal. Bioanal. Chem..

[13] Mez K., Beattie K.A., Codd G.A., Hanselmann K., Hauser B., Naegeli H., Preisig H.R. (1997). Identification of a microcystin in benthic cyanobacteria linked to cattle deaths on alpine pastures in Switzerland. Eur. J. Phycol..

[14] Chen W., Song L., Peng L., Wan N., Zhang X., Gan N. (2008). Reduction in microcystin concentrations in large and shallow lakes: Water and sediment-interface contributions. Water Res..

[15] Montagnolli W., Zamboni A., Luvizotto-Santos R., Yunes J.S. (2004). Acute effects of *Microcystis aeruginosa* from the Patos Lagoon estuary, Southern Brazil, on the microcrustacean *Kalliapseudes schubartii* (Crustacea: Tanaidacea). Arch. Environ. Contam. Toxicol..

[16] Mohamed Z.A., El-Sharouny H.M., Ali W.S.M. (2006). Microcystin production in benthic mats of cyanobacteria in the Nile River and irrigation canals, Egypt. Toxicon.

[17] Wu X., Wang C., Xiao B., Wang Y., Zheng N., Liu J. (2012). Optimal strategies for determination of free/extractable and total microcystins in lake sediment. Anal. Chim. Acta.

[18] Mohamed Z., el-Sharouny H., Ali W. (2007). Microcystin concentrations in the Nile River sediments and removal of microcystin-LR by sediments during batch experiments. Arch. Environ. Contam. Toxicol..

[19] Ihle T., Jähnichen S., Jürgen B. (2005). Wax and wane of *Microcystis* (cyanophyceae) and microcystins in lake sediments: A case study in Quitzdorf Reservoir. J. Phycol..

[20] Latour D., Salencon M.-J., Reyss J.-L., Giraudet H. (2007). Sedimentary imprint of *Microcystis aeruginosa* (cyanobacteria) blooms in Grangent reservoir (Loire, France). J. Phycol..

[21] Wörmer L., Cirés S., Quesada A. (2011). Importance of natural sedimentation in the fate of microcystins. Chemosphere.

[22] Munusamy T., Hu Y.-L., Lee J.-F. (2012). Adsorption and photodegradation of microcystin-LR onto sediments collected from reservoirs and rivers in Taiwan: A laboratory study to investigate the fate, transfer, and degradation of microcystin-LR. Environ. Sci. Pollut. Res. Int..

[23] Song H., Reichwaldt E.S., Ghadouani A. (2014). Contribution of sediments in the removal of microcystin-LR from water. Toxicon.

[24] Miller M.J., Critchley M.M., Hutson J., Fallowfield H.J. (2001). The adsorption of cyanobacterial hepatotoxins from water onto soil during batch experiments. Water Res..

[25] Wang H.X., Ho L., Lewis D.M., Brookes J.D., Newcombe G. (2007). Discriminating and assessing adsorption and biodegradation removal mechanisms during granular activated carbon filtration of microcystin toxins. Water Res..

[26] Liu G., Qian Y., Dai S., Feng N. (2008). Adsorption of microcystin LR and LW on suspended particulate matter (SPM) at different pH. Water Air Soil Pollut..

[27] Wu X., Xiao B., Li R., Wang C., Huang J., Wang Z. (2011). Mechanisms and factors affecting sorption of microcystins onto natural sediments. Environ. Sci. Technol..

[28] Arnold T.N., Oldham C.E. (1997). Trace-element contamination of a shallow wetland in Western Australia. Mar. Freshwater Res..

[29] Bailey M.C., Hamilton D.P. (1997). Wind induced sediment resuspension: A lake-wide model. Ecol. Model..

[30] Kemp A., John J. (2006). Microcystins associated with *Microcystis* dominated blooms in the southwest wetlands, Western Australia. Environ. Toxicol..

[31] Reichwaldt E.S., Song H., Ghadouani A. (2013). Effects of the distribution of a toxic *Microcystis* bloom on the small scale patchiness of zooplankton. PLoS One.

[32] Sinang S.C., Reichwaldt E.S., Ghadouani A. (2013). Spatial and temporal variability in the relationship between cyanobacterial biomass and microcystins. Environ. Monit. Assess..

[33] Ho L., Hoefel D., Saint C.P., Newcombe G. (2007). Isolation and identification of a novel microcystin-degrading bacterium from a biological sand filter. Water Res..

[34] Cao H.S., Kong F.X., Luo L.C., Shi X.L., Yang Z., Zhang X.F., Tao Y. (2006). Effects of wind and wind-induced waves on vertical phytoplankton distribution and surface blooms of *Microcystis aeruginosa* in Lake Taihu. J. Freshwater Ecol..

[35] Donati C., Drikas M., Hayes R., Newcombe G. (1994). Microcystin-LR adsorption by powdered activated carbon. Water Res..

[36] Rapala J., Lahti K., Sivonen K., Niemela S.I. (1994). Biodegradability and adsorption on lake sediments of cyanobacterial hepatotoxins and anatoxin-A. Lett. Appl. Microbiol..

[37] Lam A.K.Y., Prepas E.E., Spink D., Hrudey S.E. (1995). Chemical control of hepatotoxic phytoplankton blooms: Implications for human health. Water Res..

[38] Lambert T.W., Holmes C.F.B., Hrudey S.E. (1996). Adsorption of microcystin-LR by activated carbon and removal in full scale water treatment. Water Res..

[39] Morris R.J., Williams D.E., Luu H.A., Holmes C.F.B., Andersen R.J., Calvert S.E. (2000). The adsorption of microcystin-LR by natural clay particles. Toxicon.

[40] Grützmacher G., Wessel G., Klitzke S., Chorus I. (2010). Microcystin elimination during sediment contact. Environ. Sci. Technol..

[41] Barrington D.J., Ghadouani A., Sinang S.C., Ivey G.N. (2014). Development of a new risk-based framework to guide investment in water quality monitoring. Environ. Monit. Assess..

[42] Nang S.C.S. (2012). Spatial and Temporal Dynamics of Cyanobacteria and Microcystins in Freshwater Systems: Implications for the Management of Water Resources. Ph.D. Thesis.

[43] Yen H., Lin T., Tseng I., Tung S., Hsu M. (2007). Correlating 2-MIB and microcystin concentrations with environmental parameters in two reservoirs in South Taiwan. Water Sci. Technol..

[44] Bowman G.M., Hutka J., McKenzie N.J., Coughlan K.J., Cresswell H.P. (2002). Particle size analysis. Soil Physical Measurement and Interpretation for Land Evaluation.

[45] Heiri O., Lotter A., Lemcke G. (2001). Loss on ignition as a method for estimating organic and carbonate content in sediments: Reproducibility and comparability of results. J. Paleolimnol..

[46] Lawton L.A., Edwards C., Codd G.A. (1994). Extraction and high-performance liquid chromatographic method for the determination of microcystins in raw and treated waters. Analyst.

[47] Ghadouani A., Smith R.E.H. (2005). Phytoplankton distribution in Lake Erie as assessed by a new *in situ* spectrofluorometric technique. J. Great. Lakes Res..

[48] Beutler M., Wiltshire K.H., Meyer B., Moldaenke C., Luring C., Meyerhofer M., Hansen U.P., Dau H. (2002). A fluorometric method for the differentiation of algal populations *in vivo* and *in situ*. Photosynth. Res..

[49] (1998). APHA, AWWA, and WEF. Standard Methods for the Examination of Water and Wastewater.

